# Comparison of heart rate variability, hemodynamic, metabolic and inflammatory parameters in various phases of decompansatory hemorrhagic shock of normal and vagotomized conscious male rats

**DOI:** 10.1186/s12872-024-04342-8

**Published:** 2024-11-20

**Authors:** Fateme Khodadadi, Sujata Punait, Farzaneh Ketabchi, Zahra Khodabandeh, Aminollah Bahaoddini, Gregory F. Lewis

**Affiliations:** 1 Department of Biology, College of Sciences, Shiraz University, Shiraz, Iran; 2 Dalton Cardiovascular Research Center, Columbia, MO USA; 3 Intelligent Systems Engineering, Indiana University, The Traumatic Stress Research Consortium at the Kinsey Institute, Indiana University, Bloomington, IN United States; 4 Department of Physiology, School of Medicine, Shiraz University of Medical Sciences, Shiraz, Iran; 5 Stem Cell Technology Research Center, Shiraz University of Medical Science, Shiraz, Iran; 6 Kinsey Institute, Indiana University, Bloomington, IN United States

**Keywords:** Decompensatory shock, Heart rate variability, Subdiaphragmatic vagotomy, TNF-α

## Abstract

**Background:**

Heart rate variability (HRV) analysis has shown promise as a valuable complementary tool for clinical assessment in trauma cases. This study aims to evaluate the utility of HRV in monitoring different severities of hemorrhagic shock (HS) and its correlation with traditional hemodynamic and metabolic parameters.

**Methods:**

Male Sprague–Dawley rats were divided into different experimental groups, including those with and without vagotomy, and were exposed to different classes of decompensatory HS. To induce varying severities of HS, volume resuscitation was delayed by gradually returning 0%, 20%, or 50% of the shed blood volume at the end of the compensation phase, referred to as 0% DFR, 20% DFR, and 50% DFR class, respectively. Hemodynamic parameters were monitored, and HRV was calculated. Levels of TNF-α and IL-10 were determined in lung tissue at the end of the experiments. Correlations between HRV, hemodynamic parameters, inflammatory gene expression and arterial blood gas variables were evaluated.

**Results:**

HRV showed increased power of the low-frequency (LF) and respiratory sinus arrhythmia (RSA) in all groups during the hypotension phase of HS (Nadir 1). Subdiaphragmatic vagotomy blunted the increase in the LF component in the Nadir 1. After volume resuscitation, systolic blood pressure (SBP), RSA and LF returned to baseline in the 0% DFR and 20% DFR classes. However, animals in 50% DFR class exhibited a reduced SBP and LF and lower pH. Notably, strong correlations were found between LF and SBP as well as tissue hypoperfusion markers. The expression of TNF-α in the lung was increased in all HS groups, while this gene expression was significantly higher in the vagotomized animals.

**Conclusion:**

The alterations in HRV components were found to be significantly correlated with the hemodynamic and metabolic status of the animals, while showing no association with inflammatory responses. Additionally, the intervention of subdiaphragmatic vagotomy significantly impacted both HRV components and inflammatory responses. Collectively, these findings suggest the potential of HRV components for the assessment of the presence and severity of HS.

## Background

Trauma is one of the leading causes of death worldwide [1], and hemorrhagic shock (HS) is responsible for 30–40% of deaths from trauma [2]. In recent years, extensive efforts have been made to improve the quantity and quality of resuscitation of patients, but the mortality rate has not yet decreased [3]. Early recognition of the presence and severity of HS allows physicians to successfully treat patients [4]. However, conventional hemodynamic and metabolic parameters have long been used in the clinical evaluation of HS, which can only detect the late stages of HS [5] and lack sensitivity or specificity in the evaluation of unstable HS [6]. Therefore, the introduction of an indicator that can detect the presence of shock in the compensatory stages and its progression to higher stages will play an important role in making the right treatment decisions and reducing the mortality of patients.

Since the autonomic nervous system (ANS) drives the compensatory response to HS [5, 7], assessing ANS activity may be valuable for evaluating the severity and outcomes in patients with this condition. Research using animal models that directly recorded neural activity during HS revealed heightened neuronal activity in response to the initial loss of blood [8, 9]. Malpas & Burgess have showed that sympathetic nervous system activity (SNA) increases in the initial phase of HS and returns to baseline levels in the decompensation phase [10]. In these animal studies, changes in SNA is observed as an indication of irreversible shock [8–11]. However, the techniques for directly measuring ANS tone is invasive and not commonly used in clinical practice.

Recent studies have highlighted the potential of heart rate variability (HRV) as a noninvasive biomarker for assessing trauma patients' needs in emergency settings [12–15]. HRV refers to the variations in the time intervals between successive heartbeats, serving as an indirect measure of ANS. By analyzing these real-time variations, clinicians may gain valuable insights into a patient’s physiological state, which is essential for identifying individuals who require urgent life-saving interventions [7, 16, 17]. Various HRV measures have been explored, including frequency domain [7, 12, 16, 17], time domain [7, 13, 14, 16–18], and complexity metrics [12–14, 16, 19]. One of the most prominent components of HRV is respiratory sinus arrhythmia (RSA), which results from changes in heart rate (HR) associated with the phases of breathing. During inhalation, HR typically increases, while it decreases during exhalation, reflecting the influence of the respiratory cycle on cardiovascular function [20]. Since the parasympathetic branch of the ANS regulates interaction between the respiratory and cardiovascular systems [21], RSA serves as an indirect indicator of vagal activity [22]. The benefit of using an accurate method for estimating RSA is that the obtained values are independent of variations in respiratory parameters, such as respiratory rate and tidal volume, which can influence the measurement of cardiac vagal tone [23, 24]. Slower oscillations derived from HR analysis, known as low frequency (LF), reflect the combined activity of the SNA and the parasympathetic nervous system activity (PNA) [25]. Thus, RSA, LF, and heart period (HP, the inverse of HR) may serve as effective noninvasive biomarkers for evaluating changes in the ANS activity across different classes of HS.

There are a few studies suggesting that HRV can estimate ANS activity and detect hemorrhage in animals experiencing HS [26–28]. In addition, our recent studies have examined changes in HRV parameters in two types of compensatory [29] and prolonged classes of HS [30]. However, numerous experimental and clinical studies suggest that a major challenge in managing HS is predicting the shift from reversible decompensation to irreversible phases, a transition that can be subtle and hard to detect [31–34]. Neither our previous studies nor those of others have fully examined how HRV components vary across different severities of decompensatory HS. Therefore, the aim of the present study was to investigate the change in ANS activity (by HRV analysis) in conscious rats exposed to different classes of decompensatory HS. The correlation of HRV parameters with hemodynamic and metabolic parameters used in the clinic was evaluated to assess the feasibility of using HRV parameters as an indicator of the severity of decompensatory stages in HS. In addition, since little focus has been placed on the role of the subdiaphragmatic vagus nerve in the hemodynamic and inflammatory responses triggered by HS, this study investigated its role under these conditions.

## Methods

This study was approved by the Center for Comparative and Experimental Medicine and the Ethical Committee of Animal Care of Shiraz University of Medical Sciences, Shiraz, Iran, according to the provisions of the Declaration of Helsinki (No:IR.SUMS.MED.REC.1396.S203, Date: 03, 21, 2017). Sixty Sprague–Dawley rats (250–300 g) were purchased from the Animal- Laboratory Center of Shiraz University of Medical Sciences, Shiraz, Iran. The animals were kept under standard conditions, including a 12-h light and day cycle, a temperature of 20–22°C and humidity between 40–50%. The rats had unrestricted access to water and standard food. They were divided into eight groups: Sham (*n* = 7), vagotomized (Vag, *n* = 7), two groups with 0% delayed fluid resuscitation (0% DFR), including without (HS 0%, *n* = 6) and with vagotomy (Vag + HS 0%, *n* = 9), two groups with 20% delayed fluid resuscitation (20% DFR), including without (HS 20%, *n* = 6) and with vagotomy (Vag + HS 20%, *n* = 10) and two groups with 50% delayed fluid resuscitation (50% DFR) including without (HS 50%, *n* = 9) and with vagotomy (Vag + HS 50%, *n* = 6), each of the groups was described below.

The animals were subjected to anesthesia through an intraperitoneal injection of 50 mg/kg sodium pentobarbital (Sigma, Germany). Catheters were then inserted into the femoral vein (120-PE) and the tail artery (50 PE) and securely fastened. The surgical sites were then irrigated with 1% lidocaine (Sigma, Germany) to minimize postoperative pain in all animals. Conscious animals were housed in an optimized dark metabolic cage (MR Plexi) with their tails positioned outside the cage to allow movement without disturbing hemodynamic recordings. The arterial catheter was connected to a data acquisition system (Powerlab, PL26T04, ADinstruments, Australia) via a pressure transducer (MLT844). Arterial blood pressure was continuously monitored throughout the experiments and HR was subsequently calculated. The femoral vein catheter was used for blood sampling and blood withdrawal during induction of HS.

### Study protocol: induction of hemorrhagic shock

**Fig. 1:**
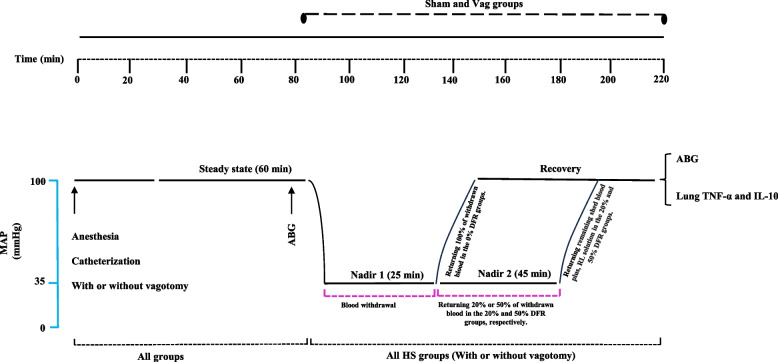
Schedule of experimental procedures in sham treatment, delayed fluid resuscitation (DFR), vagotomy (Vag), and Vag + DFR groups. The animals bled until their mean arterial pressure (MAP) reached 35 ± 5 mm Hg, which was maintained until the compensation endpoint was reached—the point at which returning the shed blood became necessary to sustain the MAP. This initial phase, referred to as Nadir 1, lasted about 25 min. After this, during the second phase called Nadir 2, which lasted about 45 min, different amounts of the maximum shed blood were returned. For the 0% DFR class, 100% of the blood was returned continuously, while for the 20% DFR and 50% DFR classes, 20% and 50% of the blood was returned gradually, respectively. Subsequently, the rats were resuscitated with the remaining blood plus 20% and 50% of the blood collected as Lactated Ringer's Solution within 10 min in the 20% DFR and 50% DFR classes, respectively. ABG: arterial blood gas analysis, MAP: mean arterial pressure, RL: Ringer's lactate

The experimental procedures are illustrated in Fig. 1. Sixty minutes after completing the surgery, 100 μl of arterial blood was collected for arterial blood gas (ABG) analysis. In the Sham group, the arterial and venous cannulas were placed in the animals without subjecting them to HS. The remaining groups were subjected to HS by withdrawing blood through the femoral vein, following established protocols from previous studies [29, 30] and our pilot experiments. Briefly, blood withdrawal began at a rate of 0.6 ml/min until mean arterial pressure (MAP) reached 35 ± 5 mm Hg. Subsequently, the blood withdrawal rate was adjusted to 0.3 ml/min to maintain MAP at the indicated level until the animal could no longer maintain its blood pressure (BP), marking the endpoint of compensation and the end of Nadir-1, which lasted approximately 25 min. In the meantime, the withdrawn heparinized blood was kept ice-cold and filtered before being reinfused to the animals. As soon as the animals' compensatory mechanisms were no longer sufficient to keep the MAP within a range of 35 ± 5 mmHg, blood withdrawal was terminated. The total blood volume of the animals was estimated based on previous studies [29, 30] and then the percentage blood loss was calculated.

Then rats were assigned to one of 3 classes of HS. Animals in the 0% DFR class, including the HS 0% and Vag + HS 0% rat groups, were resuscitated with all blood collected at the endpoint of compensation. In the other HS groups, volumes of the heparinized and filtered blood were injected continuously to maintain MAP at the level of HS. This phase was called Nadir-2. In this phase, 20% of the collected blood was returned to the 20% DFR class, including the HS 20% and Vag + HS 20% groups, or 50% of the collected blood was returned to the 50% DFR class, including the HS 50% and Vag + HS 50% groups. Nadir-2 lasted approximately 45 min in all groups.

Subsequently, resuscitation was performed by transfusion of the remaining blood plus 20% and 50% of the blood collected as Lactated Ringer's Solution within 10 min in the 20% DFR and 50% DFR classes, respectively. This phase was followed by 20 min of recording (after the resuscitation phase). Then, the arterial blood samples were taken for ABG analysis, and 1 ml of the venous blood sample was taken for determination of plasma lactate level. Finally, the animals were anesthetized with high doses of pentobarbital and their lungs were removed for molecular analysis. In the Sham group, the time courses of the experiments were the same as in the HS groups.

### Heart rate variability analysis

HRV analysis was conducted on the inter beat intervals derived from the Powerlab arterial pressure signal. Beat timing was defined as peak acceleration in pressure for each heartbeat. The approach to HRV magnitude quantification was adapted from the Porges-Bohrer method for application to the animal model used in this study [35]. HRV processing was consistent across animals and groups.

#### Sampling and smoothing

Inter beat intervals are edited using Cardio Edit for elimination of artifacts that may confound frequency-domain estimates of HRV [36]. The unevenly sampled interval data is first time sampled at 20 Hz using linear interpolation.

Next, a smooth representation of the aperiodic and slow periodic components of the HR time series is estimated from the 20 Hz time sampled IBI data. Our goal is to extract only the slow variance in interval not due to RSA and remove them without influencing the faster respiratory oscillations. We used Savitzky-Golay filter to perform smoothing as it accounts for transient effect, which is a disadvantage of moving average filters. The lower frequency content of this smoothened signal is generated by creating a 21-point cubic Savitzky-Golay filter for extraction slow aperiodic changes in the source signal below the cutoff frequency of 1.05Hz.

Similarly, to derive the LF of the IBI data; 401-point cubic Savitzky-Golay filter is applied, below the cutoff frequency of 0.05Hz. Order and frame length is selected depending on what component of signal we want to extract.

The Smoothened output is subtracted from the evenly sampled IBI data, HF 10pts each were removed at the beginning and the end and for the for LF 200 pts each.

This step is performed to eliminate biases in the estimation of RSA and LF magnitude which occurs due to ringing of filter.

#### FIR filtering

FIR filtering is performed to filter out the desired high frequency range of 1–5 Hz and lower frequency range of 0.05–0.1Hz. Filter coefficients are generated for the type 1 filter and then convolved with the difference series.

The log variance of filtered signal is calculated for discrete 30-s windows to derive the magnitude of LF in the units of Ln(ms^2^). The mean of magnitude of both the LF and RSA are calculated for further analysis.

#### Heart period

Heart period (HP) is calculated by taking the mean of the time-sampled IBIs within each 30s epoch. HP is more appropriate for deriving autonomic reactivity changes due to its linear relationship with autonomic control than the HR [37].

#### Blood pressure

Systolic (SBP) and diastolic blood pressures (DBP) were derived from the time-sampled systolic and diastolic measures for each beat, which were calculated in Powerlab. These values were further stabilized by taking the mean of 30s epoch estimates across each condition.

### Arterial blood gas parameters

The 100 μl of blood samples were taken during the baseline period and at the end of the experiments for the ABG analysis, using an easy blood gas analyzer (Medica, USA).

### Real-time PCR analysis

Tumor necrosis factor-α (TNF-α) and Interleukin 10 (IL-10) gene expression was assessed by real-time polymerase chain reaction (PCR) according to established procedures [30]. Extraction of lung RNA tissue was performed using the TriSolution plus Reagent RNA extraction kit (GeneMark, Atlanta, GA) according to the manufacturer’s guidelines. The NanoDrop Spectrometer TM (NanodropTM, Thermo Fisher Scientific, Wilmington, DE, USA) was employed to verify the quantity and purity of RNA. For cDNA synthesis, 2000 ng of RNA stored at − 80 °C was utilized, following the instructions in the cDNA Fermentas Kit (Fermentas Inc.). Primers were designed based on DNA sequences obtained from the online program Primer- BLAST of Genbank [38]. Real-time PCR was performed using the Applied BioSystems Step One ™ and the RealQ Plus 2 × Kit Master Mix Green (Ampliqon Inc); the B2M gene served as a reference for the real-time PCR reactions. The real-time PCR system was started within 10 min at 95 °C, followed by 44 cycles (15 s each at 95 °C and 60 s at 60 °C). Melting curve analysis was performed to confirm specific amplification. The results were normalized using the B2M cycle threshold (Ct), and fold change expression of TNF-α and IL-10 genes was determined using the 2^−∆∆Cq^ method.

### Statistical analysis

Features were extracted from the BP time series in custom code designed in Python. Extracted features were beat timing, SBP and DBP. Time-series representations of these features served as the basis for further signal processing within Python to extract secondary features of HRV and BP variability that served as the input to statistical analyses that were conducted in SPSS (version 29.0.2.0). One-way ANOVA was employed to test for group differences in hemodynamic parameters and inflammatory gene expression, followed by Tukey’s post hoc test for further comparisons. Two-way ANOVA with Tukey’s post hoc test was used to compare ABG parameters between the beginning and end of the experiments. Data is presented as means ± SE. Correlations were run separately for vagotomized and nonvagotomized animals and reported as Pearson r scores.

## Results

There were no differences between the groups in baseline hemodynamic parameters (Table 1). HS caused a rapid drop in MAP to 35 ± 5 mmHg, where Nadir-1 began. Blood withdrawal continued, keeping MAP around 30 ± 10 mmHg until the animals could no longer maintain this pressure, which signaled the end of Nadir-1. The duration from the beginning to the end of the Nadir-1 phase was similar in all HS and Vag + HS groups and ranged between 31.57 ± 2.8 min. Also, duration of hypotension (Nadir-1 + Nadir-2) was similar (47.2 ± 2 min) in both the 20% and 50% DFR classes.

**Table 1:** The comparison between the hemodynamic parameters at baseline of the experimental groups

	**DBP (mmHg)**	**SBP (mmHg)**	**HP (ms)**	**RSA Ln(ms**^**2**^**)**	**LF Ln(ms**^**2**^**)**
Sham	95.37 ± 2.52	125.60 ± 6.19	164.78 ± 13.48	0.088 ± 0.70	−1.77 ± 0.91
Vag	99.12 ± 5.80	121.38 ± 11.08	154.60 ± 11.49	0.15 ± 0.90	−1.54 ± 1.11
HS 0%	97.64 ± 6.60	126.26 ± 9.53	154.76 ± 18.17	0.87 ± 0.64	−2.12 ± 2.12
Vag + HS 0%	94.45 ± 5.90	123.13 ± 6.16	161.81 ± 5.12	0.57 ± 1.31	−2.27 ± 2.40
HS 20%	97.66 ± 6.82	125.59 ± 15.60	156.80 ± 17.19	0.45 ± 1.00	−1.83 ± 1.72
Vag + HS 20%	97.35 ± 6.95	127.90 ± 11.53	160.51 ± 10.17	0.08 ± 1.02	−2.48 ± 1.60
HS 50%	100.20 ± 9.76	125.91 ± 9.87	158.73 ± 12.12	0.68 ± 0.731	−1.47 ± 0.94
Vag + HS 50%	96.86 ± 5.80	129.79 ± 10.63	152.36 ± 5.67	0.12 ± 0.82	−2.01 ± 0.60

The diastolic blood pressure (DBP), systolic blood pressure (SBP), heart period (HP), respiratory sinus arrhythmia (RSA) and low frequency (LF) in the Sham (*n* = 7), Vag (*n* = 7), HS 0% (*n* = 6) and Vag + HS 0% (*n* = 9), HS 20% (*n* = 6), Vag + HS 20% (*n* = 10), HS 50% (*n* = 9) and Vag + HS 50% (*n* = 6) groups. The baseline hemodynamic parameters showed no differences between the groups. Data is mean ± SE. Comparison between groups was made with analysis of variance ANOVA with Tukey’s post hoc test

The diastolic blood pressure (DBP), systolic blood pressure (SBP), heart period (HP), respiratory sinus arrhythmia (RSA) and low frequency (LF) in the Sham (*n* = 7), Vag (*n* = 7), HS 0% (*n* = 6) and Vag + HS 0% (*n* = 9), HS 20% (*n* = 6), Vag + HS 20% (*n* = 10), HS 50% (*n* = 9) and Vag + HS 50% (*n* = 6) groups. The baseline hemodynamic parameters showed no differences between the groups. Data is mean ± SE. Comparison between groups was made with analysis of variance ANOVA with Tukey’s post hoc test.

**Fig. 2:**
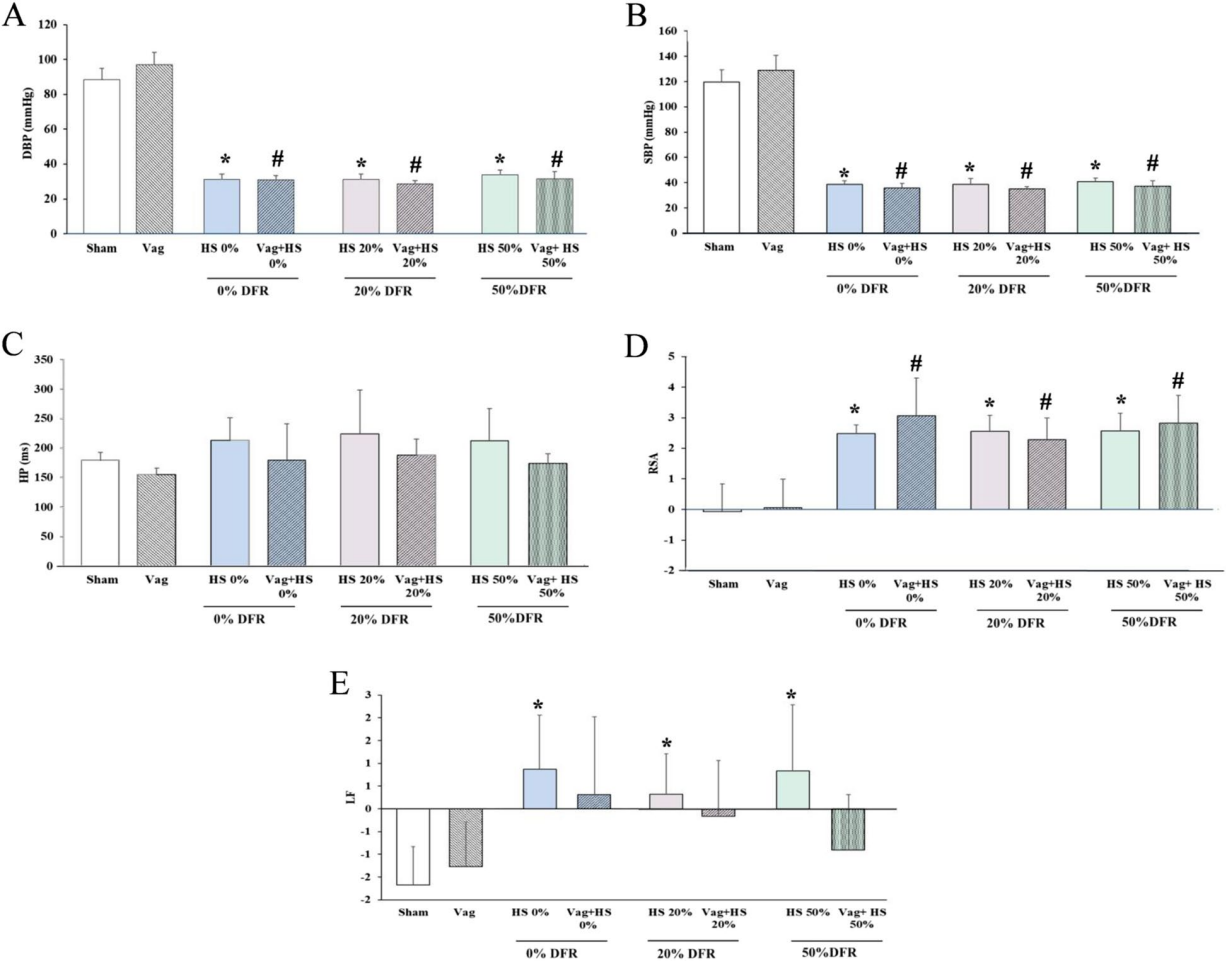
The diastolic blood pressure (DBP, A), systolic blood pressure (SBP, B), heart period (HP, C), respiratory sinus arrhythmia (RSA, D) and low frequency (LF, E) in the Sham (*n* = 7), Vag (*n* = 7), HS 0% (*n* = 6) and Vag + HS 0% (*n* = 9), HS 20% (*n* = 6), Vag + HS 20% (*n* = 10), HS 50% (*n* = 9) and Vag + HS 50% (*n* = 6) groups. Hemorrhagic shock (HS) increases both RSA and LF components, alongside a reduction in BP measurements. Vagotomy blunts the changes in LF induced by HS. Data are mean ± SE. *P < 0.05 vs. the sham group; #*p* < 0.05 vs. the Vag group. Groups were compared using ANOVA with Tukey’s post hoc test

During the Nadir-1 phase, MAP was equally maintained at 35 ± 5 mmHg in all HS groups. SBP and DBP were significantly lower in all HS and Vag + HS groups than in the Sham and Vag groups (Fig. 2). The HP, which is the time interval between heartbeats, remained relatively unchanged during this phase. However, both RSA and LF increased in all HS groups. In vagotomized animals during the Nadir-1 phase, LF did not increase significantly, while RSA did. Overall, these results indicate that, although heart rhythms may remain stable, the HRV components still change due to the effects of HS and/or vagotomy.

**Fig. 3:**
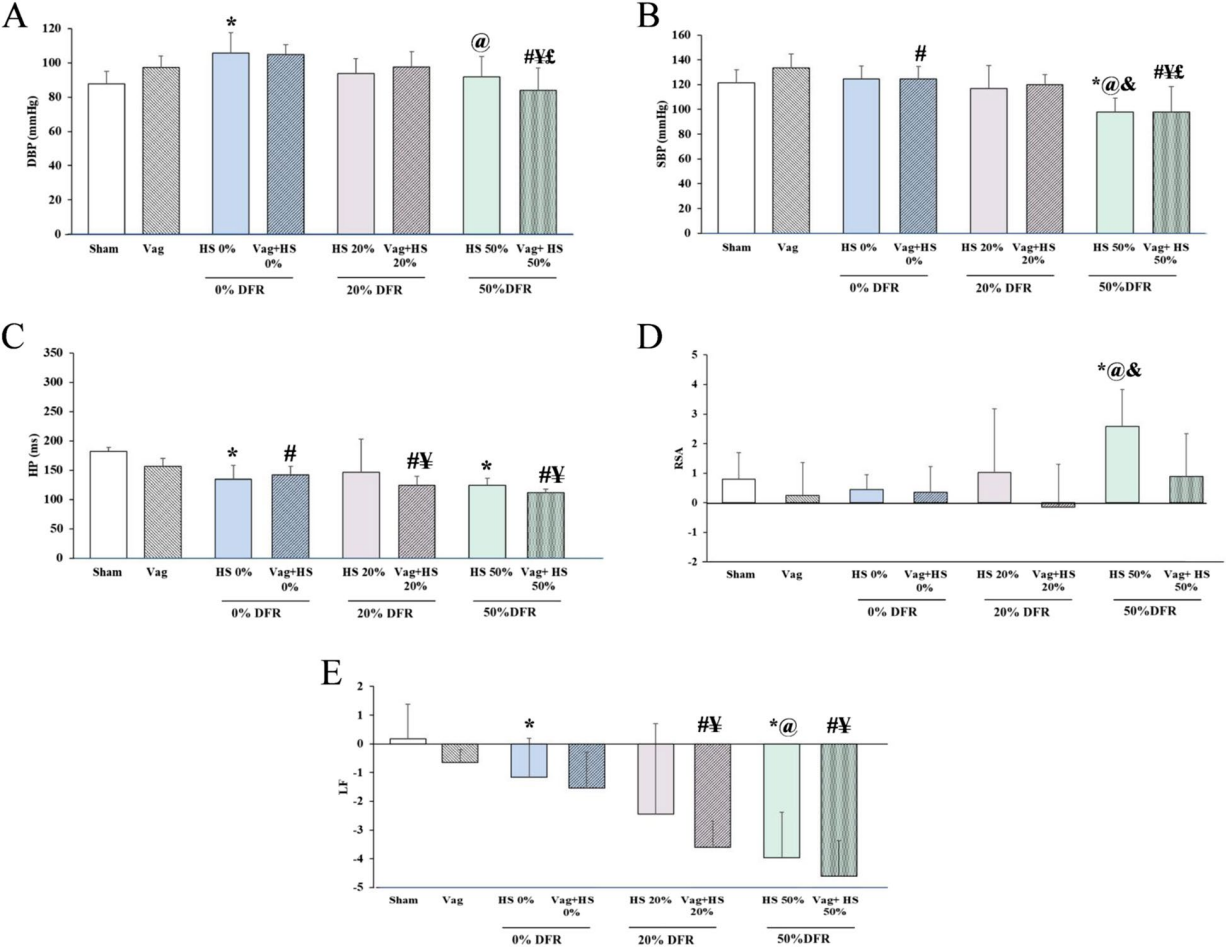
The diastolic blood pressure (DBP, A), systolic blood pressure (SBP, B), heart period (HP, C), respiratory sinus arrhythmia (RSA, D) and low frequency (LF, E) in the Sham (*n* = 7), Vag (*n* = 7), HS 0% (*n* = 6) and Vag + HS 0% (*n* = 9), HS 20% (*n* = 6), Vag + HS 20% (*n* = 10), HS 50% (*n* = 9) and Vag + HS 50% (*n* = 6) groups. Volume resuscitation restored blood pressure components (A, B), reduced HP (C), and returned RSA and LF to normal levels in most HS and Vag + HS groups. However, in the HS 50% group, the combination of low SBP, high RSA, and low LF suggests ongoing hemodynamic instability. Similar patterns of low SBP and LF were also observed in the Vag + HS 50% group. Data are mean ± SE. ^*^P< 0.05 vs. the sham group; ^#^*p* < 0.05 vs. the Vag group; ^@^*P* < 0.05 vs. the HS 0% group; ^¥^*p*< 0.05 vs. the Vag + HS 0% group; ^&^*P*< 0.05 vs. the HS 20% group; and ^£^*p*< 0.05 vs. the Vag + HS 20% group. Groups were compared using ANOVA with Tukey’s post hoc test

DBP and SBP returned to normal values in all HS groups after volume resuscitation. However, in the HS 50% group, these BP measurements gradually decreased after resuscitation and were significantly lower at the end of the experiment compared to the Sham, HS 0%, and HS 20% groups. The Vag + HS 50% group showed the same decrease in SBP and DBP, suggesting hemodynamic instability in both the HS 50% and Vag + HS 50% groups. Following volume resuscitation, HP was shorter in all HS and Vag + HS groups compared to the Sham and Vag groups, except for the HS 20% group. This indicates that the HR was higher in these groups than in the Sham and Vag groups at the end of experiments to maintain adequate blood flow. In most of the HS and Vag + HS groups, RSA was the same as in the Sham and Vag groups, suggesting that volume resuscitation restored this HRV component and brought it back to normal levels. However, in the HS 50% group, RSA remained high after volume resuscitation. After volume resuscitation, LF levels showed different patterns across the HS groups: they were higher in HS 0% compared to the Sham group, similar to the Sham group in HS 20%, and significantly lower in HS 50% than in both the Sham and HS 0% groups. Additionally, a significant drop in the LF component was seen in the Vag + HS 20% and Vag + HS 50% groups, suggesting that LF decreases in animals with hemodynamic instability (Fig. 3).

Table 2 shows the ABG measurements taken at the end of the steady state and at the end of the experiments. These parameters did not differ in all groups at the beginning of the experiment. In the HS 0%, Vag + HS 0%, and HS 20% groups, HCO3⁻ and base excess (BE) decreased significantly at the end of the experiments, but the pH levels remained within the normal range, indicating compensated metabolic acidosis in these groups. In the Vag + HS 20%, HS 50% and Vag + HS 50% groups, there was a significant decrease in tissue perfusion, as evidenced by low pH, HCO_3_^−^, and BE.

**Table 2:** Comparison of baseline and end arterial blood gas parameters, in the experimental groups

	**pHa-1**	**PaCO**_**2**_1 **(mmHg)**	**PaO**_**2**_**−1 (mmHg)**	**HCO**_**3**_**a-1 (mmol/L)**	**BE-1 (mmol/L)**	**pHa-2**	**PaCO**_**2**_**−2 (mmHg)**	**PaO**_**2**_**−2 (mmHg)**	**HCO**_**3**_**a-2 (mmol/L)**	**BE-2 (mmol/L)**
**Sham**	7.46 ± 0.00	32.78 ± 1.24	68 ± 1.12	23.75 ± 0.50	1.2 ± 0.34	7.48 ± 0.00	30.17 ± 0.91	73.24 ± 1.89	23.07 ± 0.36	0.22 ± 0.33
**Vag**	7.46 ± 0.02	32.95 ± 0.89	68.66 ± 3.84	23.55 ± 0.86	0.12 ± 1	7.48 ± 0.00	30.72 ± 1.28	68.75 ± 1.25	22.86 ± 0.65	0.12 ± 0.45
**HS 0%**	7.45 ± 0.00	33.6 ± 1.1	62.33 ± 2.67	24.46 ± 0.51	0.46 ± 0.31	7.46 ± 0.00	24.88 ± 2.69 &	65.66 ± 1.29 *	17.98 ± 1.63 &*	−4.00 ± 1.14 &*
**Vag + HS 0%**	7.47 ± 0.1	33.28 ± 1.24	63.25 ± 2.95	24.30 ± 0.76	1.36 ± 0.25	7.44 ± 0.01	27.58 ± 0.83 &	62 ± 2.04 £	19.35 ± 0.87 &#	−3.26 ± 0.90 &#
**HS 20%**	7.43 ± 0.00	35.5 ± 1.29	61 ± 2.11	24.22 ± 0.66	0.35 ± 0.44	7.45 ± 0.01	27.16 ± 1.79 &	60.7 ± 2.30	18.88 ± 0.54 &*	−3.71 ± 0.38 &*
**Vag + HS 20%**	7.44 ± 0.00	37.10 ± 1.09	67.40 ± 1.04	25.53 ± 0.70	2.1 ± 0.77	7.4 ± 0.01 &#	29.85 ± 1.12 &	67 ± 1.49	18.94 ± 1.12 &#	−4.68 ± 1.17 &#
**HS 50%**	7.48 ± 0.00	32.35 ± 0.65	62.33 ± 1.79	23.51 ± 0.37	0.71 ± 0.29	7.21 ± 0.04 &*	25.4 ± 2.53	69.2 ± 3.76	10.8 ± 1.4 &*	−15.26 ± 2–01 &*
**Vag + HS** 5**0%**	7.43 ± 0.01	36.46 ± 1.98	60.33 ± 2.66	24.96 ± 0.81	0.75 ± 0.60	7.36 ± 0.01 &#	24.13 ± 1.60 &#	66.18 ± 3.47	14.21 ± 1.19 &#	−9.05 ± 1.19 &#

Data are mean ± SE in the Sham (*n* = 7), Vag (*n* = 7), HS 0% (*n* = 6) and Vag + HS 0% (*n* = 9), HS 20% (*n* = 6), Vag + HS 20% (*n* = 10), HS 50% (*n* = 9) and Vag + HS 50% (*n* = 6) groups. Arterial blood gas analysis indicated compensated metabolic acidosis in the HS 0%, Vag + HS 0%, and HS 20% groups, while the Vag + HS 20%, HS 50%, and Vag + HS 50% groups showed uncompensated metabolic acidosis. Groups were compared using Two-way ANOVA with Tukey’s post hoc test. ^&^*P* < 0.05, versus baseline; ^*^P < 0.05, versus the Sham group; ^#^*P* < 0.05, versus the Vag group

Table 3 shows the correlation analysis between hemodynamic measurements and ABG parameters after resuscitation. The table is divided into nonvagotomized and vagotomized animals. In both vagotomized or nonvagotomized animals, there were correlations between pH, HCO_3_^−^, and BE. These metabolic parameters were correlated with two hemodynamic parameters, including SBP and LF, in all HS and Vag + HS groups indicating that lower pH is associated with reduced SBP and LF. Furthermore, SBP also correlates with DBP, RSA, and LF, suggesting SBP is linked to both metabolic and hemodynamic factors.

**Table 3:** Pearson correlation coefficient of hemodynamic and arterial blood gas parameters

NonVagotomy																																											
	**pHa-2**				**PaCO**_**2**_**−2**				**PaO**_**2**_**−2**				**HCO**_**3**_**a-2**				**BE-2**				**HP**				**LF**				**RSA**					**SBP**				**DBP**					
	r^2^		p.V		r^2^		p.V		r^2^		p.V		r^2^		p.V		r^2^		p.V		r^2^		p.V		r^2^		p.V		r^2^		p.V		r^2^			p.V		r^2^			p.V		
**pHa-2**	1.000																																										
**PaCO**_**2**_**−2**	−0.046		0.823		1.000																																						
**PaO**_**2**_**−2**	−0.120		0.559		−0.374		0.060		1.000																																		
**HCO**_**3**_**a-2**	.751**		0.000		.592**		0.001		−0.216		0.290		1.000																														
**BE-2**	.911**		0.000		0.349		0.080		−0.183		0.372		.956**		0.000		1.000																										
**HP**	0.358		0.072		0.279		0.168		0.163		0.425		.489*		0.011		.475*		0.014		1.000																						
**LF**	.505**		0.009		0.232		0.254		0.244		0.229		.587**		0.002		.596**		0.001		.752**		0.000		1.000																		
**RSA**	-.406*		0.040		0.101		0.623		−0.121		0.557		−0.311		0.122		−0.365		0.067		−0.051		0.795		−0.155		0.432		1.000														
**SBP**	.504**		0.009		0.183		0.370		−0.011		0.958		.516**		0.007		.538**		0.005		0.305		0.114		.461*		0.013		-.506**		0.006		1.000										
**DBP**	0.044		0.833		0.075		0.716		−0.118		0.565		0.025		0.902		0.031		0.881		−0.297		0.125		−0.049		0.804		0.053		0.790		.582**			0.001		1.0					
**Vagotomy**																																											
		**pHa-2**				**PaCO**_**2**_**−2**				**PaO**_**2**_**−2**				**HCO**_**3**_**a-2**				**BE-2**				**HP**				**LF**				**RSA**					**SBP**					**DBP**			
		r^2^		p.V		r^2^		p.V		r^2^		p.V		r^2^		p.V		r^2^		p.V		r^2^		p.V		r^2^		p.V		r^2^		p.V			r^2^		p.V			r^2^		p.V	
**pHa-2**		1.000																																									
**PaCO**_**2**_**−2**		.452*		0.018		1.000																																					
**PaO**_**2**_**−2**		−0.071		0.742		−0.290		0.191		1.000																																	
**HCO**_**3**_**a-2**		.804**		0.000		.887**		0.000		−0.283		0.202		1.000																													
**BE-2**		.886**		0.000		.805**		0.000		−0.268		0.227		.987**		0.000		1.000																									
**HP**		.610**		0.000		0.354		0.070		−0.090		0.674		.549**		0.003		.590**		0.001		1.000																					
**LF**		.684**		0.000		0.364		0.062		0.086		0.689		.597**		0.001		.657**		0.000		.763**		0.000		1.000																	
**RSA**		0.013		0.948		−0.085		0.672		0.180		0.401		−0.075		0.709		−0.074		0.714		0.018		0.923		0.002		0.993		1.000													
**SBP**		.449*		0.015		0.309		0.116		0.120		0.575		.394*		0.042		.407*		0.035		.529**		0.002		.632**		0.000		-.412*		0.019			1.000								
**DBP**		0.231		0.229		0.123		0.541		0.141		0.511		0.167		0.404		0.178		0.375		0.288		0.110		.436*		0.013		−0.146		0.424			.662**		0.000			1.000			

All HS and Vag + HS groups showed correlations between pH, HCO_3_^−^, and BE with SBP and LF in all HS and Vag + HS groups. Additionally, SBP correlated with DBP, RSA, and LF. The Pearson correlation coefficient (r^2^); and *p* value (p.V); ^*^p < 0.05; ^**^p < 0.001

**Fig. 4:**
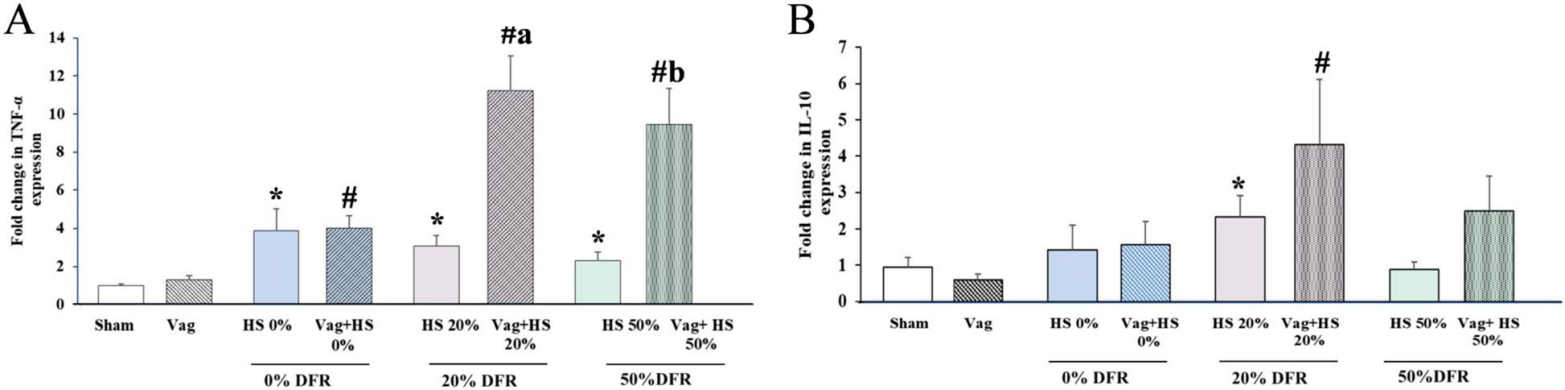
The expressions of TNF-α (**A**) and IL-10 (**B**) in lung in the Sham (*n* = 7), Vag (n = 7), HS 0% (*n* = 6) and Vag + HS 0%(*n* = 9), HS 20%(*n* = 6), Vag + HS 20%(*n* = 10), HS 50%(*n* = 9) and Vag + HS 50%(*n* = 6) groups. Data means ± SE. TNF-α gene expression in the lungs was elevated in all hemorrhagic shock (HS) groups, and vagotomy further increased this expression in the 20% and 50% DFR classes (A). IL-10 expression significantly increased only in the 20% DFR class. The comparison between groups was performed by using parametric one-way ANOVA and Tukey’s post hoc multiple-comparison test. ^*^P < 0.05, versus the Sham group; ^#^*P* < 0.05, versus the Vag, group; ^a^*P* < 0.05, versus the HS 20% and ^b^*P* < 0.05, versus the HS 50%

Gene expressions of TNF-α in the lungs were elevated in all HS groups, regardless of whether vagotomy was performed. Interestingly, vagotomy further increased TNF-α expression in the 20% DFR and 50% DFR classes (Fig. 4A). Significant increases in IL-10 expression were observed only in the HS 20% and Vag + HS 20% groups (Fig. 4B). The failure to upregulate IL-10 in the lungs in 50% DFR class may be linked to organ dysfunction in these animals [39]. There was no correlation between the expression of these inflammatory genes and hemodynamic parameters in nonvagotomized animals, suggesting that the inflammatory responses in these animals may not be linked to their hemodynamic status. In contrast, in vagotomized animals, TNF-α expression negatively correlated with hemodynamic indicators like HP and LF, indicating that increased inflammation may be linked to reduced LF and HP. Overall, these results suggest that vagotomy not only increases inflammatory gene expression but also changes how inflammation relates to hemodynamics (Table 4).

**Table 4:** Pearson correlation coefficient of hemodynamic and inflammatory gene expression

**Nonvagotomy**												
	**IL-10**		**HP**		**SBP**		**DBP**		**LF**		**RSA**	
	r^2^	p.V	r^2^	p.V	r^2^	p.V	r^2^	p.V	r^2^	p.V	r^2^	p.V
**TNFa**	.469^*^	0.032	−0.067	0.749	0.131	0.534	0.317	0.123	−0.108	0.608	−0.066	0.752
**IL10**			−0.095	0.673	0.162	0.470	0.309	0.162	−0.050	0.826	0.122	0.589
**HP**					0.305	0.114	−0.297	0.125	.752^**^	0.000	−0.051	0.795
**SBP**							.582^**^	0.001	.461^*^	0.013	-.506^**^	0.006
**DBP**									−0.049	0.804	0.053	0.790
**LF**											−0.155	0.432
**Vagotomy**												
	**IL-10**		**HP**		**SBP**		**DBP**		**LF**		**RSA**	
	r^2^	p.V	r^2^	p.V	r^2^	p.V	r^2^	p.V	r^2^	p.V	r^2^	p.V
**TNFa**	.624^**^	0.006	-.634^**^	0.001	−0.282	0.181	−0.023	0.914	-.529^**^	0.008	−0.030	0.890
**IL10**			−0.440	0.068	−0.224	0.372	0.052	0.839	−0.465	0.052	-.511^*^	0.030
**HP**					.529^**^	0.002	0.288	0.110	.763^**^	0.000	0.018	0.923
**SBP**							.662^**^	0.000	.632^**^	0.000	-.412^*^	0.019
**DBP**									.436^*^	0.013	−0.146	0.424
**LF**											0.002	0.993

In contrast to nonvagotomized animals, vagotomized animals showed a significant correlation between TNF-α expression and hemodynamic indicators such as HP and LF. Pearson correlation coefficient (r^2^); and *p* value (p.V); ^*^p < 0.05; ^**^p < 0.001

## Discussion

HS is one of the leading causes of death worldwide, causing damage to various organs, including the lungs. In treatment units, vital signs are used to detect shock; however, they can only identify it during decompensatory stages [40, 41]. In these stages, a lack of appropriate treatment can cause HS to progress to sever level, leading to hemodynamic collapse, metabolic acidosis, and further drops in BP, ultimately resulting in organ dysfunction and death [42]. Many studies indicate that predicting the severity of HS and its development into more severe stages is challenging, which complicates management [31–34]. Therefore, an index that provides real-time information on the presence and quantification of HS severity is crucial for improving outcomes. This gap in knowledge motivated us to investigate whether HRV components could serve as reliable indicators of HS severity. Furthermore, their correlation with other hemodynamic, metabolic, and inflammatory parameters has been evaluated. Additionally, the influence of subdiaphragmatic vagotomy on hemodynamic, as well as metabolic and inflammatory responses to different severities of HS, was evaluated.

In this study, we developed a well-quantified model of HS in conscious animals that delineates the progression of HS to more severe classes, driven by delays in volume resuscitation. The finding of the present study demonstrated that HRV components changed significantly across different stages and severities of HS, and these changes were linked to SBP and the animals' metabolic status. These findings suggest that HRV components may serve as real-time indicators of HS severity. Regarding the use of HRV to estimate ANS activity, the changes in HRV components indicate that ANS activity varied across different stages of HS. In the Nadir 1 phase, the autonomic balance was predominantly parasympathetic across all HS groups. After volume resuscitation, PNA returned to baseline levels in animals with normal BP, while it remained elevated in animals experiencing hemodynamic instability. This suggests that PNA restoration may indicate overall recovery and hemodynamic stability after HS. TNF-α expressions in the lung increased after HS followed by resuscitation. Subdiaphragmatic vagotomy blunted the changes LF component during HS and exacerbated HS-increased TNF-α expressions in lung suggesting that subdiaphragmatic vagus nerve activity effects low variations in HRV and proinflammatory responses. Taken together, these results suggest that HRV parameters may indicate the presence and severity of HS and that subdiaphragmatic vagotomy effects on HRV components and proinflammatory responses.

No changes in BP, HRV parameters and ABG variables were observed in any of the groups at the start of the experiments. Therefore, all groups start the study under identical conditions. Furthermore, these results indicate that unilateral subdiaphragmatic vagotomy during the steady-state phase has no effect on the above parameters [29, 30].

In the Nadir 1 phase of the HS group, MAP was kept in the lowest possible range, similar to other studies in animals subjected to HS [11, 30]. Low BP measurements in this phase were associated with increases in RSA and LF. RSA is an indicator of PNA and LF is an indicator that reflects influence of both the SNA and PNA [25]. The increase in the parasympathetic component of HRV during the Nadir 1 phase of HS was also observed in our previous studies [29, 30], suggesting that the autonomic balance shifts in favor of PNA as blood withdrawal continues. These studies showed that during parasympathetic hyperactivity, the LF band also increases in parallel with the increase in the parasympathetic band. Therefore, the higher LF band in the Nadir 1 phase of the HS group might be related to hyperparasympathetic activity [43]. Hyperparasympathetic activity during the Nadir 1 phase may occur as a response to rapid and significant blood withdrawal. Oberg and Thoren first demonstrated that rapid blood loss activates mechanoreceptors in the left ventricle, increasing the discharge of unmyelinated vagal fibers, which leads to bradycardia [44]. This response, known as the Bezold-Jarisch reflex, occurs when more than 20% of blood volume is lost. The reduction in blood volume increases heart contractility and HR, activating stretch-sensitive receptors. These receptors then send signals to the brainstem, inhibiting SNA and enhancing PNA [27, 45]. This hyperparasympathetic response may serve as a defense mechanism, reducing BP to promote clot formation and prevent further bleeding [46]. Overall, the increase in both RSA and LF during the Nadir 1 phase of HS may result from hyperparasympathetic activity and could serve as an indicator of shock presence.

Although RSA increased during the Nadir 1 phase in the Vag + HS groups, vagotomy blunted the LF increase, possibly reflecting impaired autonomic nerve responses, consistent with our previous findings [29]. Supporting our results, earlier studies demonstrated that the abdominal vagus nerve plays a significant role in ANS activity, and that abdominal vagotomy disrupts this function [47].

In the Nadir 2 phase, the MAP in the 20% and 50% DFR classes was maintained at the same level as in the Nadir 1 phase. Although the amount of blood withdrawn and the duration of the Nadir-2 phase were identical in the 20% and 50% DFR classes, the HS 50% and Vag + 50% HS groups required more returned blood (50% of blood withdrawn) to remain in this phase, indicating severe hemodynamic instability in these animals. However, due to the additional effects of fluid injection on HR, we did not consider HR and HRV at this stage. Future studies are needed to further characterize the reasons and mechanisms of the different compensatory responses in 20% vs 50% DFR classes to the same amount and duration of blood withdrawn.

After volume resuscitation, SBP and DBP returned to baseline in the 0% DFR and 20% DFR classes. However, in the 50% DFR class, these BP measurements gradually decreased after resuscitation and were significantly lower at the end of the experiment compared to other classes, suggesting the 50% DFR class experienced hemodynamic instability. The RSA was similar in the most HS groups to the Sham group suggesting that volume resuscitation restored this HRV component and brought it back to normal levels. However, this component was still higher in the HS 50% group than the Sham group. The reduction in SBP with high RSA suggests that the animals in the HS 50% group may still be in shock despite receiving volume resuscitation. In the Vag + HS 50% group, even though the animals had low SBP, their RSA levels were similar to those in the Vag group, suggesting that interruption of the vagus by subdiaphragmatic vagotomy supports recovery of vagal tone [29]. Interestingly, the LF component decreased with the delay of resuscitation and was significantly lower in the 50% DFR class, which exhibited hypotension. A lower LF with unchanged RSA may be due to a disruption in SNA [18, 30]. Overall, these results suggest that the restoration of HRV components after volume resuscitation may indicate hemodynamic stability. In contrast, a higher RSA component and a lower LF component may be associated with hemodynamic instability after HS followed by resuscitation.

The delay in resuscitation caused the compensatory responses to fail and a decompensated state developed, leading to inadequate tissue perfusion, as evidenced by a decrease in pH and a lower BE and bicarbonate levels in the arterial blood. In the 0% and 20% DFR classes, although arterial bicarbonate and BE decreased, the pH remained around normal. This indicates that the compensatory mechanisms were effective in managing the hypoperfusion caused by HS. However, pH, bicarbonate, and BE significantly reduced in the 50% DFR class indicating metabolic acidosis in this class as a result of disruption of tissue perfusion, and anaerobic metabolism. In this model, HS severity was better indicated by the percentage of shed blood volume returned than by the duration of hypotension. This approach showed variability in physiological responses between animals. For instance, while the 20% and 50% DFR classes had the same duration of hypotension, hypoperfusion was much more severe in the 50% DFR class, as seen by changes in ABG parameters. This reflects differences in animals' compensatory and decompensatory responses [34]. Furthermore, these results show that the model of HS used in this study is a reliable tool for observing and understanding how HS progresses to more severe stages in conscious animals. Interestingly, there was a significant correlation between these metabolic parameters with LF, suggesting that the lower LF component of HRV may be a reliable index of hemodynamic and metabolic disorder.

Several studies have investigated the role of the vagus nerve in inflammation in recent years. Some articles suggest that adenosine, lactate, ROS and cytokines released after HS increase the sensitivity of the vagal afferent fibers of the lung [48]. These fibers are mainly C-fibers that trigger protective reflexes such as cough modulation, bronchial contraction, mucus secretion [48] and cholinergic anti-inflammatory [49, 50]. However, other studies have shown that vagus nerve health is critical for increased cytokine and inflammatory effects and vagotomy reduces the production of pro-inflammatory cytokines and inhibits fibrogenic cell function [51]. Therefore, there is no consensus on the inflammatory and anti-inflammatory effects of the vagus nerve.

We recently demonstrated that vagotomy exacerbates disruption of the alveolar-capillary barrier and increases lung tissue inflammation in Intermediate class of HS [29]. In the present study, HS followed by resuscitation increased the expression of TNF-α in the lung, which is consistent with our previous study [30]. Subdiaphragmatic vagotomy exacerbated TNF-α gene expression in lung tissue. These results are consistent with our previous study reporting that vagotomy increased lung tissue inflammation in HS [29], indicating an anti-inflammatory effect of the vagus [52]. However, in another article, subdiaphragmatic vagotomy resulted in a reduction in the expression of these proinflammatory cytokines [30], demonstrating the role of vagal activation in an anti-inflammatory response. The different experimental conditions could play a role in these different results. Further studies are needed to uncover the role of the vagus nerve in inflammatory responses in HS. In contrast to the biochemical markers of hypoperfusion, the inflammatory markers showed a weak correlation with the HRV parameters. This weak correlation could be due to the difference in the neuroanatomy of the fibers involved in the regulation of HR and inflammation [53].

## Limitation

One potential limitation of this study was the small sample size of animals. However, based on the sample size (N) in each group, we estimate a 95% chance of detecting larger effects and an 80% chance of finding smaller effects with a size of d = 0.83. Additionally, hemodynamic and HRV parameters were monitored and calculated for a short duration (20 min) following resuscitation. If this observation period had been extended to 60 min, it’s likely that the differences in cardiovascular and HRV parameters would have been more pronounced. However, the one-hour survival rates were low for the animals in the HS 50% and Vag + HS 50% groups, which were 0% and 10%, respectively. Furthermore, HRV components can be influenced by anesthesia, as anesthetic drugs reduce all components of HRV [54]. To mitigate this issue, HS was induced 90 min after the administration of sodium pentobarbital, ensuring that the animals were awake. If any residual effects of the anesthetic drugs remained on the HRV parameters, they would be consistent across all groups of animals.

## Conclusion

The heart rate variability components exhibited significant changes across various stages and severities of hemorrhagic shock. These changes were linked to the hemodynamic and metabolic status of the animals, but not to their inflammatory responses. Subdiaphragmatic vagotomy influenced HRV components both during and after resuscitation, as well as pro-inflammatory responses. These findings suggest that HRV components could serve as real-time indicators of HS severity.

## Data Availability

The datasets supporting the conclusions of this article are included within the article.

## Abbreviations

BP: Blood pressure
DBP: Diastolic blood pressure
SBP: Systolic blood pressure
MAP: Mean arterial blood pressure
HS: Hemorrhagic shock
Vag: Sub-diaphragmatic vagotomized
Vag + HS: Sub-diaphragmatic vagotomized with hemorrhagic shock
DFR: Delayed fluid resuscitation
HP: Heart period
HR: Heart rate
HRV: Heart rate variability
LF: Low frequency
RSA: Respiratory sinus arrhythmia
ANS: Autonomic nervous system
SNA: Sympathetic nervous system activity
PNA: Parasympathetic nervous system activity
TNF-α: Tumor necrosis factor-α
IL-10: Interleukin 10
ABG: Arterial blood gas analyzing
PCR: Polymerase chain reaction
PaO_2_: Arterial venous oxygen pressure
PaCO_2_: Arterial carbon dioxide pressure
BE: Base excess
Ct: Cycle threshold
RL: Ringer's lactate
